# Hereditary cancer syndromes with gynecological cancer risk: focus on prevention strategies

**DOI:** 10.3389/fonc.2026.1811964

**Published:** 2026-04-28

**Authors:** Simona Duranti, Valentina Iacobelli, Rita Trozzi, Floriana Camarda, Arianna Panfili, Anna Fagotti, Francesco Fanfani, Claudia Marchetti, Camilla Nero

**Affiliations:** 1 Scientific Directorate, Fondazione Policlinico Universitario Agostino Gemelli, IRCCS, Rome, Italy; 2 Unit of Oncological Gynecology, Women’s Children’s and Public Health Department, Fondazione Policlinico Universitario Agostino Gemelli, IRCCS, Rome, Italy; 3 Università Cattolica del Sacro Cuore, Rome, Italy; 4 UOC Genetica Medica, Fondazione Policlinico Universitario Agostino Gemelli, IRCCS, Rome, Italy

**Keywords:** *BRCA1/2*, genetic counseling, hereditary cancer syndrome, Lynch syndrome, preventive strategies, prophylactic surgery, surveillance

## Abstract

**Background:**

Hereditary cancer syndromes, including pathogenic variants in *BRCA1/2* and mismatch-repair genes, confer a substantial risk of several malignancies, including ovarian, endometrial, and fallopian tube cancers. Given the limited efficacy of current screening strategies, particularly for ovarian cancer, a prevention-focused approach is required. This review synthesizes evidence on identification, risk stratification, surveillance, chemoprevention, and prophylactic surgery in women with inherited gynecologic cancer susceptibility, proposing a precision-prevention framework.

**Methods:**

A structured search of MEDLINE, Embase, and the Cochrane Library was conducted through July 2025. Original studies, reviews, and guidelines in English were included following independent screening and full-text assessment by two authors.

**Results:**

Expanded germline testing, universal mismatch-repair screening, and genomic profiling have improved carrier identification beyond family history–based criteria. Integrated counseling models enhance informed decision-making and access to care. Conventional surveillance tools show limited sensitivity; emerging strategies, including circulating tumor DNA assays and artificial intelligence, require further validation. Hormonal and anti-inflammatory agents demonstrate potential for risk reduction. Prophylactic surgery, including salpingo-oophorectomy, hysterectomy, or investigational salpingectomy with delayed oophorectomy, remains central, requiring multidisciplinary evaluation and attention to fertility, menopausal health, and patient preferences. Ethical and health-economic considerations remain critical in clinical practice and policy development. Further studies are warranted to better elucidate the potential role of liquid biopsy, microbiota, and targeted vaccination strategies.

**Conclusions:**

Prevention of gynecologic cancers in genetically predisposed women requires an integrated strategy that includes comprehensive genetic assessment, risk-adapted surveillance, evidence-based risk-reduction interventions, and multidisciplinary coordination. Implementing and refining precision prevention frameworks is crucial to optimize outcomes and translate genetic risk into tailored preventive care.

## 1 Introduction

Women who are carriers of pathogenic variants (PVs) associated with hereditary cancer syndromes (HCS), such as *BRCA1/2* and mismatch repair (MMR) PVs, have a substantially elevated lifetime cancer risk. Concerning gynecological cancers, women with *BRCA1* PVs may have up to a 58% lifetime risk of epithelial ovarian cancer, and *BRCA2* carriers of up 29% (1). Those with Lynch syndrome confront a 40–60% risk of endometrial cancer and increased risk for OC, depending on the gene involved, comparable to the general population for *PMS2*, and up to 38% for PVs in *MSH2 (*2, 3). PVs in other genes are also associated with an increased risk of developing gynecological cancer, albeit lower than *BRCA* and Lynch syndrome genes.

Despite advances in screening and treatment, gynecologic cancers, particularly OC, are frequently diagnosed at advanced stages, resulting in substantial morbidity and mortality. An improved understanding of the genetic basis of these malignancies is therefore central to informing prevention-focused approaches and optimizing outcomes in high-risk populations. In the setting of HCS, where lifetime cancer risks are well characterized and evidence-based risk-reduction strategies exist, prevention represents a key component of clinical management. The rationale for emphasizing preventive strategies lies in their demonstrated potential to reduce cancer incidence and mortality, particularly for cancers such as OC, in which early detection remains limited due to nonspecific symptoms and the lack of effective screening tools.

This review aims to assess the estimated risk of gynecologic malignancies associated with major hereditary cancer predisposition syndromes in order to inform evidence-based surveillance and risk-reduction strategies, guided by current clinical guidelines and supported by the latest scientific literature.

## 2 Methods

**Figure 1:**
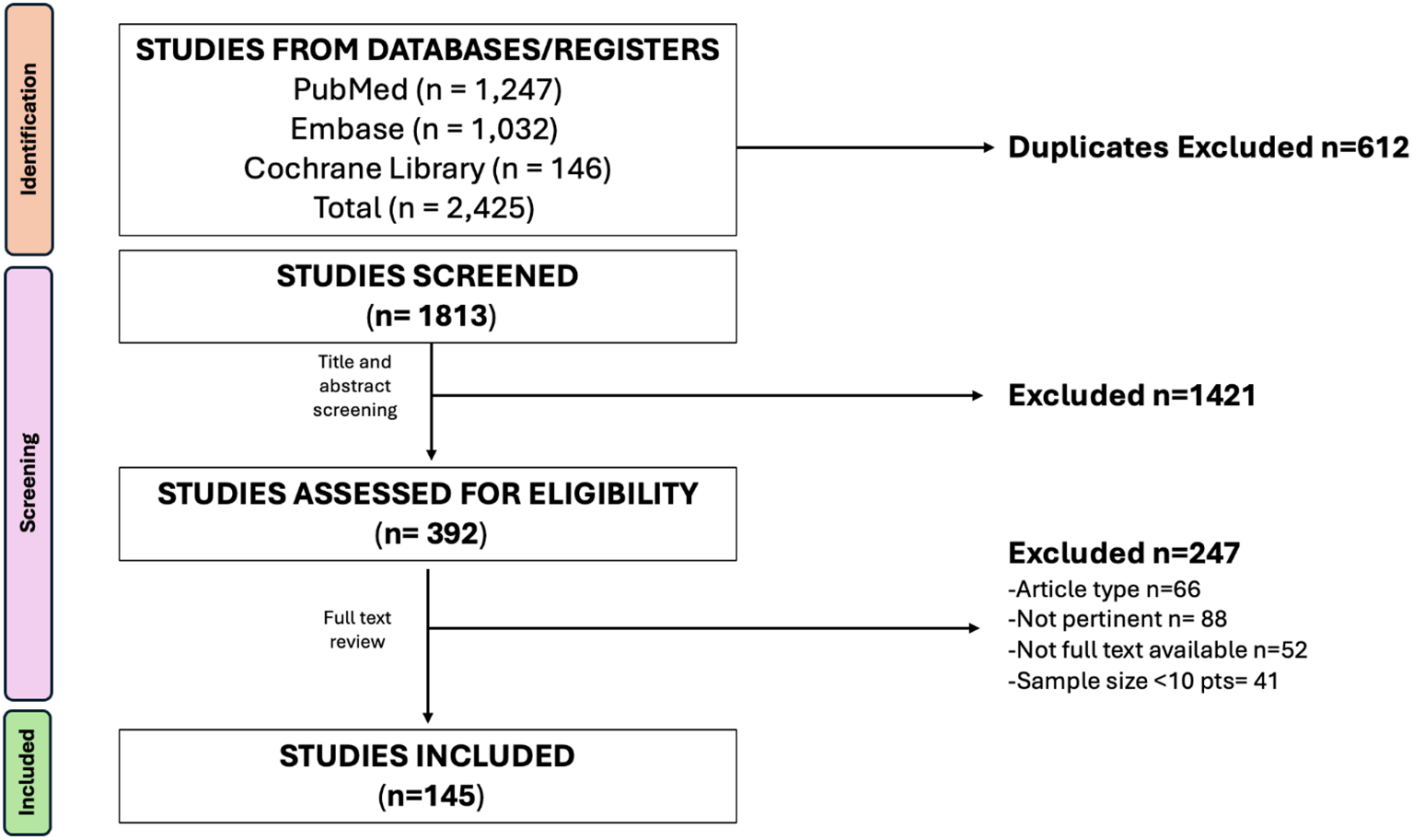
Flow diagram of the study selection process.

This review was conducted as a structured narrative review to enable a critical synthesis of heterogeneous evidence encompassing epidemiological studies, clinical trials, guideline statements, and translational research on gynecologic cancer prevention in HCS. A narrative approach was considered appropriate given the variability in study designs, outcomes, and gene-specific penetrance estimates, which precluded formal quantitative meta-analysis. Methodological quality and relevance were assessed qualitatively using principles derived from the Scale for the Assessment of Narrative Review Articles (SANRA) (4), focusing on: justification of review design, transparency of literature search, scientific reasoning, appropriate referencing, and balanced interpretation of evidence. A systematic literature search was performed in MEDLINE (via PubMed), Embase, and the Cochrane Library, with the final search executed on July 15, 2025. The PubMed search strategy combined controlled vocabulary (MeSH terms) and free-text keywords, including: “hereditary cancer syndromes” OR “BRCA1” OR “BRCA2” OR “Lynch syndrome” OR “mismatch repair genes” AND “gynecologic cancer” OR “ovarian cancer” OR “endometrial cancer” AND “risk” OR “surveillance” OR “risk-reducing surgery” OR “prophylactic surgery” OR “chemoprevention”. No restrictions on publication date were applied. The search was limited to English-language articles. The PubMed search yielded 1,247 records, while Embase identified 1,032 records and the Cochrane Library 146 records, for a total of 2,425 records. After removal of 612 duplicates, 1,813 unique records were screened. Two reviewers (S.D. and V.I.) independently screened titles and abstracts, excluding 1,421 records that were not relevant to the scope of the review. A total of 392 articles underwent full-text assessment for eligibility. Of these, 247 studies were excluded for the following reasons: non pertinent (n=88), insufficient sample size (<10 patients; n=41), not full text available (n=52), or article type (n=66). Ultimately, 145 studies were included in the final qualitative synthesis, comprising original research articles, systematic reviews, meta-analyses, and international guideline statements. Discrepancies between reviewers were resolved through discussion and consensus. Although a formal PRISMA framework was not applied due to the narrative design, the study selection process followed a structured and reproducible approach (**Figure 1**). Findings were synthesized narratively, prioritizing high-quality evidence, large cohort studies, meta-analyses, and consensus guideline recommendations (e.g., ESGO, NCCN, ESMO), with particular attention to clinically actionable prevention strategies.

## 3 Epidemiology and genetic basis

HCS are estimated to affect approximately 2–5% of the general population, with prevalence varying according to the specific genetic condition and demographic characteristics (5). These syndromes are typically associated with germline PVs, mostly in high-penetrance cancer susceptibility genes. This estimate includes well-characterized syndromes such as Lynch syndrome, Hereditary Breast and Ovarian Cancer (HBOC) syndrome, Li-Fraumeni syndrome, and Familial Adenomatous Polyposis (FAP), among others.

The incidence and lifetime risk of gynecologic malignancies vary significantly across HCS, with *BRCA1/2* and Lynch syndrome- related PVs representing the most prevalent and clinically significant. Accurate gene-specific risk assessment is essential for developing tailored surveillance and prophylactic strategies in genetically predisposed individuals.

Women carrying *BRCA1* variants face a lifetime risk of epithelial OC ranging from 39% to 58%, while *BRCA2* carriers have a lower but still elevated risk of approximately 13% to 29%, with the highest incidence between 50–59 years and 60–69 years for *BRCA1* and *BRCA2*, respectively (6–11). Endometrial cancer risk is not markedly increased in *BRCA* PVs carriers, though emerging data suggest a possible association for serous endometrial cancer in *BRCA1* individuals (12, 13). Regarding breast cancer risk, *BRCA1* gene is associated to 60-72% lifetime risk, while *BRCA2* gene to 55-69% (10, 14). Moreover, constitutional PVs in *BRCA* genes are also associated to an increased lifetime risk of pancreatic cancer both for women and men and prostate cancer in men (15–17).

Lynch syndrome, caused by germline PVs in mismatch repair genes such as *MLH1, MSH2, MSH6*, and *PMS2*, confer a 40–60% lifetime risk of endometrial cancer and a 10–12% risk of OC, often presenting at a younger age and with distinct histopathological features. Moreover, *EPCAM* deletions of the 3’ end of the gene are associated to Lynch syndrome, since they cause *MSH2* gene hypermethylation and its subsequent silencing (18). The lifetime risk of gynecologic cancers varies substantially depending on the gene involved, delineating four distinct syndromic entities within the spectrum of Lynch syndrome (2, 3, 19, 20). Indeed, among MMR genes, *MLH1* and *MSH2* PVs confer the highest lifetime risks: 34-54% and 21-57% for endometrial cancer and 4-20% and 8-38% for OC, respectively. *MSH6* PVs are associated with a 16–49% lifetime risk of endometrial cancer and a ≤1-13% lifetime risk of OC, often with later onset. *PMS2* PVs carry a comparatively lower risk, with lifetime estimates around 13–26% for endometrial cancer and 1.3-3% for OC, while some studies suggesting no significant increase in ovarian cancer risk for *PMS2* gene. For *EPCAM* gene deletions the gynecological cancer risk depends on site and location of deletion (18, 21). Lynch syndrome is also associated to an increased risk of other cancer types, in particular gastrointestinal (colorectal, gastric, pancreatic, biliary tract) and genitourinary cancers (2, 3, 19).

Beyond *BRCA1/2* and MMR, other genes are implicated in gynecologic HCS. In particular, the following genes are associated with an increased ovarian cancer lifetime risk: *ATM* (2-3%), *PALB2* (3-5%), *BRIP1* (5-15%) and *RAD51C* and *RAD51D* (10-15% and 10-20%, respectively) (22–29). Moreover, other HCS, including Cowden syndrome (*PTEN* PVs), Peutz–Jeghers syndrome (*STK11* PVs) and Li–Fraumeni syndrome (*TP53* PVs), are associated with relevant risk of gynecologic cancers, particularly endometrial and cervical neoplasms. In particular *PTEN* gene is associated to a 6-22% increased risk of EC (30, 31), *STK11* is associated to an increased risk of endometrial cancer (10%), cervical cancers (10%, usually adenocarcinoma) and ovarian cancer (10%, in particular sex cord tumor with annular tubules, SCTAT) (32, 33), while *TP53* is rarely linked to gynecologic cancers, but may predispose to early-onset uterine sarcomas (34). Also *MUTYH*-associated polyposis (MAP) seems to be related to an increased risk of endometrial cancer (35, 36), and this risk appears increased in germline PVs *POLE* and *POLD1* carriers (37). Importantly, germline PVs in the *DICER1* gene, represents a rare but increasingly recognized HCS characterized by a broad spectrum of benign and malignant neoplasms. Although its prevalence is low, *DICER1* alterations are particularly relevant in gynecologic oncology due to their association with specific tumor types, including ovarian Sertoli–Leydig cell tumors, gynandroblastomas, and other sex cord–stromal tumors, often occurring at a young age (38). While the absolute lifetime risk of gynecologic malignancies in *DICER1* carriers remains incompletely defined, current evidence supports its inclusion among HCS with distinct histopathologic and clinical features that may inform diagnosis, surveillance, and genetic counseling (38).

## 4 Risk assessment and genetic testing

The cornerstone of precision prevention is early identification of women with inherited PVs in cancer predisposing genes. Genetic counseling ensures informed decision-making, accurate family risk assessment, and tailored management plans (39). Testing should be offered not only to affected individuals, but also to other at-risk relatives through cascade testing.

The paradigm of hereditary cancer risk assessment is rapidly evolving, driven by the implementation of universal screening for MMR deficiency (40), the expansion of germline testing criteria, and the integration of comprehensive genomic profiling in cancer centers’ diagnostic algorithms (41–43). These advancements enable the identification of individuals carrying PVs beyond the limitations of traditional pedigree-based algorithms that often fail to capture sporadic or atypical presentations.

Genetic counseling constitutes an integral component of the germline genomic testing continuum and is recommended by professional societies, including the National Comprehensive Cancer Network (NCCN) and the American College of Medical Genetics and Genomics (ACMG) (44), as a prerequisite to and requisite follow-up for hereditary cancer testing. Counseling is conventionally delivered through structured pre-test and post-test consultations: these encounters address both the medical implications of germline findings and their associated psychosocial sequelae, including anxiety, decisional conflict, and survivor guilt (39).

Contemporary counseling frameworks incorporate a standardized, multidisciplinary approach encompassing patient education, psychosocial evaluation, and individualized risk assessment. Pre-test counseling includes discussion of test indications, gene content, analytic validity, clinical sensitivity and limitations, potential result categories, downstream implications for medical management, cascade testing, and familial risk communication. Post-test counseling focuses on the interpretation of results according to gene-specific variant classification criteria within the context of personal and family history, and supports guideline-concordant decision-making regarding surveillance, prophylactic interventions, cascade testing of at-risk relatives, and referral to appropriate subspecialty care and support services (39). Post-test genetic counseling should also address reproductive implications, including the risk of transmission to offspring, typically 50% for autosomal dominant conditions, and the available reproductive options. These may include prenatal diagnostic procedures (e.g., chorionic villus sampling or amniocentesis) and preimplantation genetic testing for monogenic disorders (PGT-M) in the context of assisted reproduction, which enables the selection of embryos not carrying the familial variant (45, 46). Such counseling should be delivered by experienced clinical geneticists, with careful consideration of ethical, psychological, and socio-cultural aspects, and with respect for the values and principles guiding patient care.

In this context, genetic testing and cancer prevention strategies raise significant ethical, legal, and social considerations that must be integrated into clinical practice. These include respect for patient autonomy, informed consent, confidentiality, and the right to control the use and disclosure of genetic information (47, 48).

As genetic testing becomes increasingly accessible, particularly through the expansion of telehealth and alternative delivery models, the integration of robust counseling protocols and equitable testing criteria is essential to maximize clinical utility while minimizing potential harm. It is critical that counseling frameworks evolve in parallel with technological advancements to uphold ethical standards and optimize patient outcomes in precision medicine. To support this transition toward broader and more inclusive genetic evaluation, innovative counseling models have emerged, such as tele-genetics (49), mainstreaming genetic testing into routine oncology care (50), and cascade testing within families (51). These models have demonstrated effectiveness in enhancing patient engagement, facilitating informed consent, and improving adherence to germline testing recommendations. Moreover, they help address systemic barriers to care, promoting equitable access across geographically and socioeconomically diverse populations (52–54). Ensuring equitable access to genetic services and culturally sensitive counseling remains crucial, as disparities in access and underrepresentation in genomic databases may limit the accuracy of variant interpretation and the overall clinical utility of testing.

Accurate identification of individuals with gynecologic HCS is essential for defining effective risk-reduction strategies and implementing precision prevention approaches. According to guidelines published by NCCN and European Society for Medical Oncology (ESMO), germline genetic testing should be offered to women with a personal history of high-grade epithelial ovarian, fallopian tube, or primary peritoneal cancer, with a endometrial cancer diagnosed under age 50 or with mismatch repair deficiency (dMMR), a family history suggestive of HBOC or Lynch syndrome, a known pathogenic variant in the family, or Ashkenazi Jewish ancestry (regardless of family history) (1, 55, 56). Recent expansions of the NCCN criteria now support consideration of genetic testing in unaffected individuals with close relatives who meet the specified criteria, reflecting a shift toward more inclusive, risk-informed care. In addition, the widespread adoption of large next-generation sequencing panels for somatic tumor profiling has prompted the development of recommendations for reflex testing when suspected germline variants are identified (41).

Receiving genetic testing can elicit complex emotional responses. Evidence indicates that individuals with pathogenic results frequently experience increased short-term anxiety, whereas negative results may be associated with survivor guilt (39, 57). In addition, results involving variants of uncertain significance (VUS) can generate persistent uncertainty and psychological distress (48, 57). Factors influencing the psychological impact of genetic testing include personal and familial cancer history, individual risk perception, and the availability of social support networks. A subset of patients may experience decisional regret, particularly when testing is undertaken without adequate pre-test preparation. Incorporating structured psychological assessment tools into both pre- and post-test counseling may help identify individuals at risk of adverse emotional outcomes and mitigate these effects (58, 59). According to current international guidelines and ethical recommendations, genetic testing for *BRCA* and Lynch syndrome is generally advised after the age of 18, in order to ensure the individual’s capacity for autonomous and informed decision-making, including the right not to know. Exceptions are restricted to rare circumstances involving families with very early-onset cancers (approximately 17–18 years). In contrast, for genes associated with a high risk of severe, early-onset malignancies (e.g., S*TK11, TP53, PTEN*), genetic testing is typically recommended during childhood, in line with established guidelines, with informed consent obtained from both parents or legal guardians.

Ethical challenges in genetic testing primarily concern respect for individual autonomy, confidentiality, and the duty to warn. Upholding an individual’s right to decline testing must be carefully balanced against the potential health implications for biologically related family members. In addition, the disclosure of incidental or secondary findings remains ethically contentious, reflecting ongoing debate regarding patients’ rights to know or not know such information (60). Additionally, concerns regarding genetic discrimination in insurance and employment persist, despite the implementation of legislative protections in several countries (61). Ethical frameworks must also promote equitable access to genetic testing, particularly for marginalized and underserved populations, with respect to resource allocation, and address key considerations related to testing in minors, reproductive decision-making, and the ownership, governance, and use of genetic data (62).

## 5 Primary prevention strategies

Once a HCS has been identified and cancer risk assessed by a geneticist, carriers should be managed by appropriate specialists to define personalized prevention programs. Genetic re-evaluations are also recommended to update surveillance and risk-reduction programs, or to discuss with patients who have not yet opted for prophylactic surgery the risk–benefit balance, as well as the potential role of chemoprevention according to the most recent guidelines. The periodic re−assessment of variants of uncertain clinical significance is equally crucial for individuals who carry them.

Consultation with a fertility specialist may also be warranted, particularly for *BRCA* carriers, as some studies suggest an earlier onset of menopause and accelerated oocyte aging (63). Moreover, such evaluations are especially relevant given the young age at diagnosis of cancer or precancerous lesion, such as complex atypical hyperplasia in Lynch syndrome, which may further compromise reproductive potential, considering also the lower efficacy of fertility sparing strategy (64, 65).

Primary prevention in women with inherited predisposition to gynecologic cancers focuses on interventions that reduce cancer incidence before disease onset. These include prophylactic surgery and chemoprevention, and both of which must be tailored to the individual’s genetic profile, age, reproductive plans, comorbidities and patients’ preference.

### 5.1 Prophylactic surgery

Given the lack of effective screening and early detection methods associated with OC, risk-reducing salpingo-oophorectomy (RRSO) is considered the most effective strategy for preventing ovarian, fallopian tube, and primary peritoneal cancers in *BRCA* carriers (7, 66, 67). Meta-analyses have reported an 80–85% reduction in the risk of these cancers, along with a significant decrease in all-cause mortality, particularly when the procedure is performed prior to natural menopause (68, 69). The timing of the procedure varies depending on the specific gene involved. For *BRCA1* carriers, surgery is typically recommended between the ages of 35 and 40, or after childbearing is complete. In contrast, *BRCA2* carriers may delay the procedure until ages 40 to 45, due to the later onset of associated cancer risks (10, 56, 70). However, also after RRSO, 1-4.3% risk of primary peritoneal carcinoma residual risk is reported in some studies (71, 72). Additionally, a reduction in breast cancer risk is reported; however, the evidence remains inconclusive and appears to differ depending on the specific *BRCA* variant (73, 74). Routine hysterectomy is not universally recommended in *BRCA* carriers, but may be considered on an individual basis, particularly in *BRCA1* carriers given the reported increased risk of serous endometrial carcinoma, or to safely allow the use of hormone replacement therapy after RRSO (70). Preoperative evaluation before prophylactic surgery should include serum CA-125 testing and pelvic ultrasound (1). Salpingectomy has been shown to reduce the risk of ovarian cancer in general population and could be an option in women with HCS in premenopausal age (75). In this setting, to reduce the consequences of early menopause, the TUBA-WISP II (76), the PROTECTOR (77) and the SOROCk (78) trials, currently ongoing, are investigating whether salpingectomy with delayed oophorectomy is a safe and effective alternative to standard RRSO in women at high inherited risk of ovarian cancer (B*RCA1, BRCA2, BRIP1, RAD51C, RAD51D*, and *PALB2* carriers).

Considering the elevated lifetime risk of developing endometrial cancer in Lynch syndrome, particularly among carriers of *MLH1, MSH2*, or *MSH6* PVs, prophylactic total hysterectomy combined with bilateral salpingo-oophorectomy is recommended following the completion of childbearing (79, 80). The timing of this preventive intervention is typically considered between the ages of 35 and 45 (generally after 40 years), with adjustments based on gene-specific penetrance and individual’s family history (55). The procedure offers substantial clinical benefits, including near-complete elimination of endometrial cancer risk. Additionally, it contributes to a reduction in ovarian cancer incidence, especially in individuals with *MLH1* and *MSH2* variants, since the ovarian cancer risk in *MSH6* variants is lower and similar to the general population for *PMS2*. Accordingly, NCCN guidelines recommend hysterectomy with opportunistic salpingectomy starting from age 40 in *MSH6* carriers, with delayed oophorectomy around age 50, whereas in carriers of *PMS2* PVs, hysterectomy with RRSO may be considered from 50 years (55). Considering the high risk of incidental findings of cancer during prophylactic surgery, up to 17% based on recent literature, a pre-operative assessment should be performed, including transvaginal ultrasound and endometrial sampling (81).

Considering other ovarian cancer predisposition genes, RRSO is recommended between the ages of 45 and 50 for carriers of PVs in *PALB2, BRIP1, RAD51C*, and *RAD51D (*1). However, there is currently insufficient evidence to support a recommendation for *ATM* carriers. For Cowden and Peutz Jeghers syndromes, the option of hysterectomy should be considered upon completion of childbearing (55). No data are currently available regarding prophylactic surgery in individuals with *DICER1* variants.

**Figure 2:**
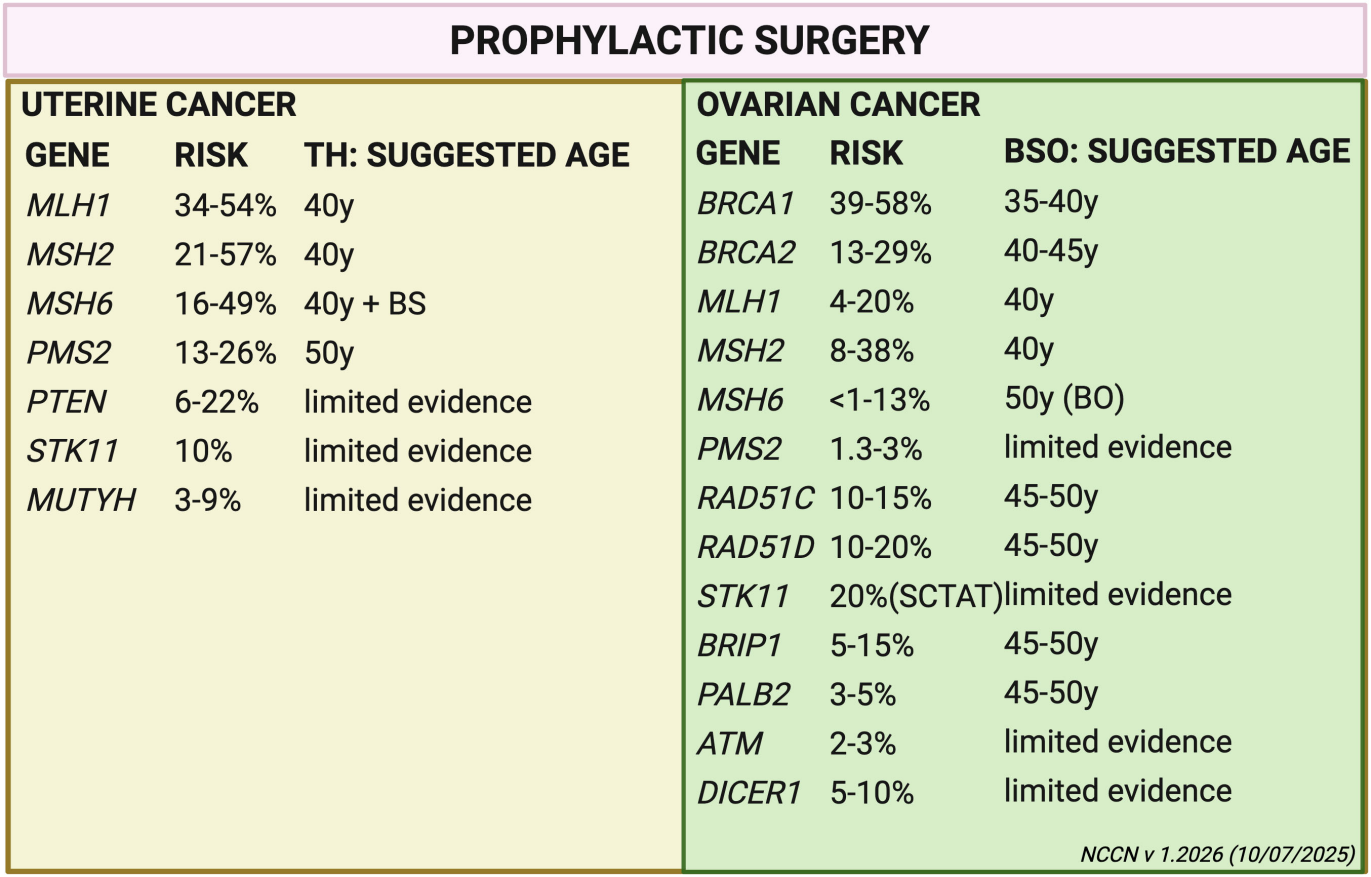
This figure represents gene-specific lifetime risks of uterine and ovarian cancer and the corresponding recommended age for prophylactic surgery. TH, total hysterectomy; BSO, bilateral salpingo-oophorectomy; BS, bilateral salpingectomy; BO, bilateral oophorectomy; SCTAT, sex cord tumor with annular tubules.

Lifetime risks and recommendations for RRSO are summarized in **Figure 2**.

Menopausal status and quality-of-life considerations are central to clinical decision-making, particularly in the context of prophylactic surgical interventions. Despite its oncologic benefits, prophylactic surgery induces menopause, characterized by an abrupt loss of estrogen. This hormonal deprivation is associated with increased risks of osteoporosis, cardiovascular disease, cognitive decline, and sexual dysfunction. To mitigate these adverse effects, hormone replacement therapy (HRT) is generally recommended until the average age of natural menopause (approximately 51 years) in women without contraindications. In the setting of HCS, the use of HRT must be carefully balanced against potential oncologic risks, highlighting the need for individualized counseling and further research to clarify its long-term safety and impact (82–85).

### 5.2 Chemoprevention

#### 5.2.1 Contraceptives

Available evidence consistently demonstrates that oral contraceptive (OCP) use is associated with a significant reduction in ovarian cancer risk in *BRCA1* and *BRCA2* PVs carriers, estimated at approximately 50% (86–91). In contrast, an increased risk of breast cancer has been reported, particularly with prolonged exposure; however, findings across studies are heterogeneous and conflicting (92). Several analyses suggest that the inverse association between OCP use and ovarian cancer risk may be stronger than the potential positive association with breast cancer risk, resulting in a net long-term reduction in the combined risk of breast, ovarian, and endometrial cancers. Nonetheless, given that the cumulative risk of breast by age 50 exceeds that of ovarian cancer and continues to increase with age, the clinical balance between benefits and risks of OCP use remains uncertain, also at younger ages when ovarian cancer risk is relatively low (87, 88).

Concerning long-acting reversible contraceptives (LARC) progestin-based and cancer risk, evidence is limited, particularly in *BRCA1/2* PVs carriers. In the general population, intrauterine device (IUDs) and injectable progestins are associated with reduced ovarian cancer risk, with an increase in breast cancer risk (93–98). Limited data in *BRCA1/2* carriers suggest a possible protective effect of LARC on ovarian cancer risk, but the evidence is sparse, and further studies are needed (99). Some evidence both from the general population and *BRCA* variants carriers suggests a protective effect of combined OCPs and IUDs on ovarian cancer risk, increasing with duration of use (86, 95, 100).

Regarding Lynch syndrome, evidence on the use of hormonal oral contraceptives and IUD is limited. Available data do not suggest an increased risk associated with hormonal contraception compared with the general population. However, the impact of hormonal contraceptives use on endometrial cancer risk reduction remains controversial, with retrospective studies reporting conflicting results and prospective trials showing reduced endometrial proliferation but no data on endometrial cancer incidence (101, 102). Concerning levonorgestrel-releasing IUDs, most data are extrapolated from the general population, where a substantial reduction in endometrial cancer risk of about 50% has been demonstrated; however, evidence on Lynch syndrome is inconclusive (95). Data on ovarian cancer risk in Lynch syndrome carriers are lacking.

#### 5.2.2 Aspirin

In the context of HCS, especially Lynch syndrome, aspirin has emerged as a promising chemopreventive agent. Long-term observational and interventional studies have demonstrated that regular aspirin use is associated with a significant reduction in colorectal cancer incidence and mortality, particularly in individuals with MMR gene variants. Aspirin’s anticancer activity is thought to be mediated through multiple mechanisms, in particular antiplatelet effects, through the inhibition of platelet activation, thereby reducing the release of pro-tumorigenic growth factors and cytokines, anti-inflammatory action, inhibiting cyclooxygenase enzymes and reducing chronic inflammation (103–106). In Lynch syndrome, the CaPP2 trial demonstrated that high-dose aspirin (600 mg/day) significantly reduced colorectal cancer risk after long-term follow-up. However, efficacy in reducing the risk of gynecological neoplasms has not been demonstrated (107). Recently, the CaPP3 trial showed that low-dose aspirin (75–100 mg daily) was found to be just as effective as higher doses in reducing the risk of colorectal cancer (108). Specific gynecological outcomes have not yet been reported in the published results.

Regarding OC, previous studies support a possible role of aspirin in reducing ovarian cancer risk in general population. However, a large-scale randomized trial involving 40,000 healthy female health professionals from the Women’s Health Study, found that alternate-day low-dose aspirin in healthy women did not reduce the incidence of ovarian cancer (109). Other randomized controlled trials showed a decreased risk of female reproductive cancers when aspirin is used for at least 3 years for cardiovascular prevention and meta-analyses, pooled analyses of case control studies and prospective studies have found that aspirin may reduce ovarian cancer risk by 10%-20% when used daily and by 34-44% with low dose continuous long-term use (110–117). In *BRCA1/2* carriers, a randomized phase II placebo-controlled trial (STICs and STONEs; NCT03480776) is currently ongoing to evaluate whether aspirin reduces the risk of ovarian and fallopian tube cancer, including its potential impact on the detection of serous tubal intraepithelial carcinomas (STICs) at the time of prophylactic surgery.

Other antiplatelet agents are under investigation, but aspirin remains the most studied and widely endorsed due to its safety profile and affordability. However, individual risk assessment is essential, as aspirin use may increase the risk of gastrointestinal bleeding, particularly in older adults.

## 6 Secondary prevention strategies

Secondary prevention strategies in women carrying germline PVs focus on surveillance and early detection, though their efficacy in reducing mortality remains limited.

For OC, screening modalities such as serum CA-125 measurement and transvaginal ultrasound are commonly employed. However, these tools suffer from low sensitivity and specificity, and do not reduce the risk of developing or dying from tubo-ovarian carcinoma; for this reason, they should not be offered as alternative to prophylactic surgery in carriers of ovarian cancer susceptibility genes, but are recommended for preoperative planning (1). Two trials specifically addressed these issues. The UK Familial ovarian cancer Screening Study evaluated the role of annual transvaginal ultrasound and CA125 testing in 3,563 women at high risk of OC, including HCS carriers, achieved a sensitivity of 81.3% for incident ovarian cancer detection, with positive and negative predictive values of 25.5% and 99.9%, respectively. Only 30.8% of screen-detected cancers were diagnosed at stage I–II, but not screened women significantly presented with advanced-stage disease (≥IIIC) (118). The subsequent phase II of the trial evaluated ROCA-based CA125 screening in 4,348 women at high risk of OC. Patients were screened with CA125 interpreted using the ROCA algorithm every 4 months, and annual transvaginal ultrasound if ROCA results were normal, or within 2 months if ROCA results indicated intermediate risk. Screening showed high sensitivity and negative predictive value and it was associated with a significant stage shift and higher rates of optimal cytoreduction; however, it was associated with a low positive predictive value. No conclusions on survival benefit could be drawn, as the study was not designed to assess these outcomes (119).

In the context of Lynch syndrome, endometrial cancer screening has not proven benefit, since current evidence does not support a significant reduction in cancer-related mortality, but may facilitate early detection of endometrial abnormalities. Indeed, endometrial biopsy is both highly sensitive and specific and can be considered every 1–2 years starting from 30–35 years (55). Transvaginal ultrasound has not shown to be sufficiently sensitive nor specific, especially in premenopausal setting due to variability in endometrial thickness, but may be considered at the clinician’s discretion (55).

The same recommendations provided for endometrial surveillance can also be extended to Cowden syndrome (1).

**Figure 3:**
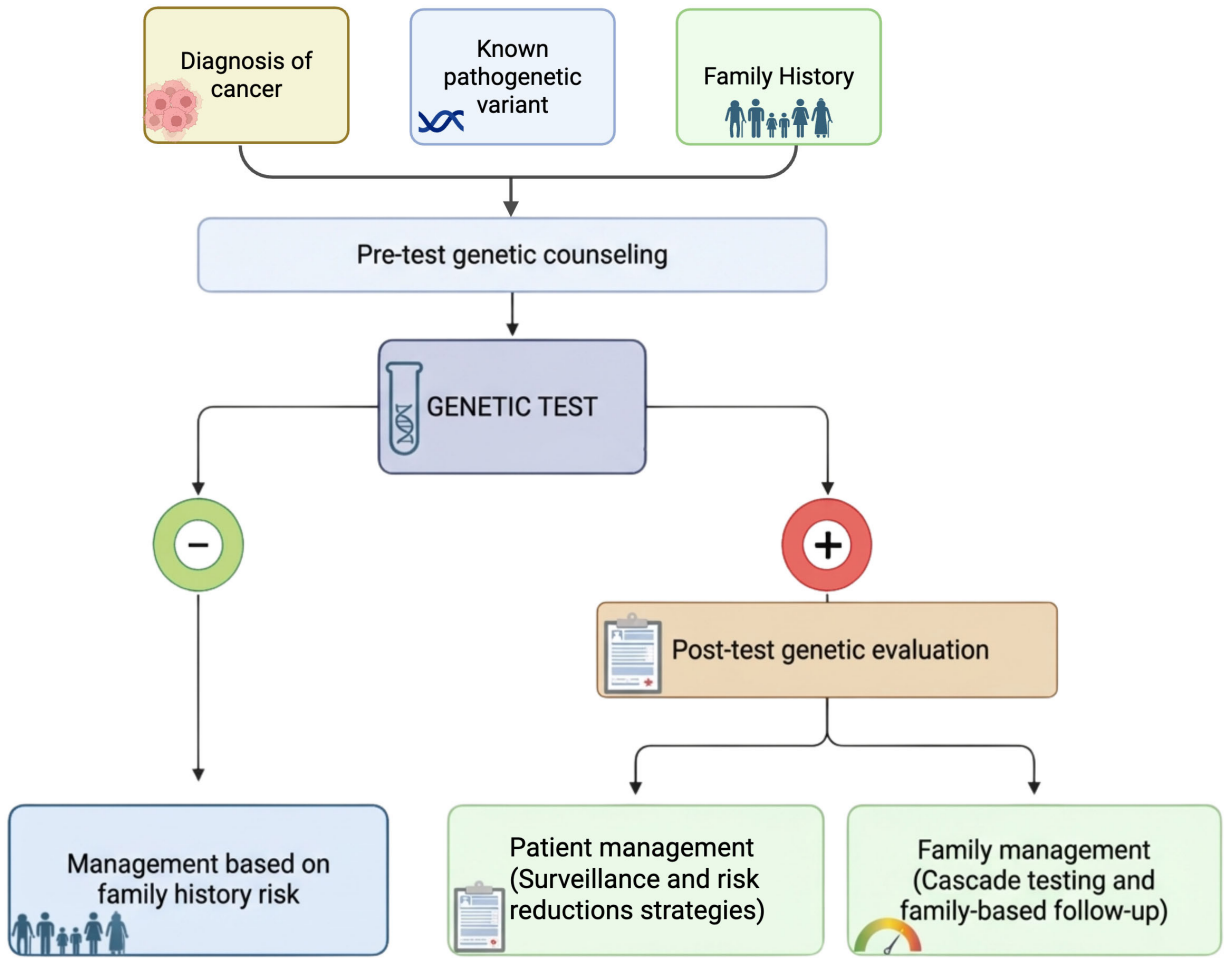
Genetic testing is indicated based on tumor type, family history, or identification of a germline pathogenic variant in a relative. Detection of a germline variant guides surveillance, risk-reduction strategies, and cascade testing, whereas uninformative results require management based on family history.

The workflow for identifying HCS and defining surveillance and risk-reduction strategies is summarized in **Figure 3**.

## 7 Lifestyle and behavioral modifications

Evidence on the influence of lifestyle factors on cancer risk in individuals with HCS is emerging but remains limited.

Concerning *BRCA* carriers, existing research suggests that modifiable behaviors may play a role. A systematic review reported insufficient evidence to support tailored dietary or weight−management recommendations specifically for ovarian or breast cancer risk reduction in *BRCA1/2* carriers beyond general population guidance, with some data suggesting that higher diet quality, adulthood weight loss, and physical activity during adolescence or young adulthood might be associated with reduced breast cancer risk (120). Observational studies indicate that obesity, smoking, and alcohol consumption are common among *BRCA* carriers and may be associated with increased breast cancer risk, while physical activity and healthy weight are associated with lower risk or prevalence (121, 122). Prospective lifestyle intervention trials such as the LIBRE study, currently ongoing, aim to evaluate the effect of diet and physical activity on metabolic and fitness outcomes, though evidence on cancer incidence remains forthcoming (123).

In Lynch syndrome carriers, several observational studies and systematic reviews suggest that modifiable lifestyle factors such as body weight, physical activity, and diet may influence cancer risk, although high-quality prospective data are limited (124–127). A systematic review found that overweight/obesity and adult weight gain may be associated with increased colorectal cancer risk, while physical activity and higher fruit intake may be associated with reduced risk, mirroring trends observed in the general population (115). Moreover, in the CAPP2 trial, long-term follow-up showed that daily supplementation with resistant starch significantly reduced the incidence of non-colorectal Lynch syndrome–associated cancers, particularly upper gastrointestinal cancers, without affecting colorectal cancer risk (128). Recommendations for weight control and physical activity that apply broadly to cancer prevention may also be relevant for Lynch syndrome carriers, but robust syndrome-specific evidence is lacking. Additionally, an international cross-sectional survey highlighted substantial gaps in adherence to general lifestyle guidelines (e.g., diet, exercise, alcohol) among individuals with MMR genes PVs, underscoring potential targets for behavioral interventions (129).

Collectively, these findings support the hypothesis that lifestyle modification could serve as an adjunctive strategy for cancer prevention in hereditary risk populations, but further prospective cohort studies and intervention trials are needed to clarify the magnitude and causality of these associations.

## 8 Novel approaches

In the context of HCS, emerging technologies such as liquid biopsy, vaccine development and artificial intelligence (AI) are reshaping early detection and risk stratification strategies.

Liquid biopsy enables the non-invasive analysis of circulating tumor DNA (ctDNA), offering a powerful tool for surveillance beyond traditional imaging and tissue biopsy techniques and holding promise for at-risk individuals with germline cancer predispositions syndromes. Liquid biopsy has already demonstrated potential utility in monitoring and non-invasive early cancer detection, including evidence that multimodal ctDNA assays can identify cancer-associated signals prior to conventional clinical diagnosis (130–132). Indeed, aberrant DNA methylation is one of the earliest and most stable epigenetic changes during carcinogenesis, making methylation profiling a particularly valuable biomarker for early cancer detection. Circulating cell-free DNA methylation assays have shown promise for non-invasive early cancer detection across multiple cancer types and could complement or improve upon mutation-based ctDNA approaches, especially in scenarios where ctDNA levels remain low in early or preclinical disease stages (133–135). In individuals with HCS the integration of methylation profiling of ctDNA into surveillance protocols could potentially facilitate earlier identification of neoplastic changes ahead of clinical presentation, supporting personalized prevention strategies. Although large-scale validation in longitudinal cohorts is still needed, the accumulating evidence highlights the transformative potential of biomarkers in HCS management (136).

In parallel, immunopreventive vaccination is emerging as a novel strategy for cancer interception in Lynch syndrome, as its associated neoplasms accumulate recurrent frameshift mutations that generate shared, highly immunogenic neoantigens. Nous-209, a viral vector–based vaccine encoding 209 shared frameshift peptides, was evaluated in a phase 1b/2 single-arm trial in healthy LS carriers (NCT05078866). The vaccine demonstrated a favorable safety profile with no treatment-related serious adverse events and induced neoantigen-specific T-cell responses in 100% of evaluable participants, with broad and durable immunity detectable up to one year. Functional analyses confirmed both CD8^+^ and CD4^+^ T-cell activation and cytotoxic activity against neoantigen-expressing targets. Although not powered to assess cancer incidence reduction, exploratory colonoscopic findings showed no advanced adenomas or colorectal cancers at end-of-study evaluation, supporting the biological plausibility of immune interception (137). Larger randomized studies are planned to determine its preventive clinical impact.

AI-driven predictive models further enhance the utility of these biomarkers by integrating complex genomic and epigenomic data, improving diagnostic accuracy, and supporting robust risk stratification. Machine learning and deep learning approaches applied to multi-omics datasets can uncover subtle patterns not easily detected through conventional analyses, paving the way for earlier and more individualized prediction of disease onset and progression. AI has also been highlighted as a method for driving advanced epigenomic analytics in pan-cancer detection pipelines. However, ethical and implementation challenges remain prominent (138). The integration of AI in genomic diagnostics raises concerns related to data privacy, algorithmic transparency, informed consent, and equitable performance across diverse populations. These ethical considerations are particularly acute when predictive models are applied to asymptomatic individuals, necessitating clear communication strategies and consent processes that reflect the complexities of AI-assisted risk assessment (139).

Moreover, some evidence suggest the gut microbiota could play a key role in cancer development (140, 141), in particular some evidence showed that Colibactin-producing Escherichia coli is implicated in colorectal carcinogenesis (142, 143). In hereditary syndrome, however, its role is less defined. Since not all individuals with HCS, in particular with Lynch syndrome, develop cancers, germline variants alone are insufficient to explain disease onset, suggesting a synergistic interaction between host genetics and the gut microbiome (144, 145). Further research, also in gynecological cancers, is required to elucidate the contribution of the gut microbiota to cancer development and prevention.

Finally, preliminary evidence also supports the feasibility of detecting genomic instability directly from Papanicolaou/endocervical samples as an early marker of ovarian carcinogenesis. In a proof-of-principle study by Paracchini, Marchini and colleagues, low-pass whole-genome sequencing of DNA obtained from archival Pap test samples identified copy-number profile abnormalities in women who subsequently developed high-grade serous ovarian cancer (HGSO, with detectable signals up to 9 years before diagnosis; the resulting EVA test showed a sensitivity of 75% and a specificity of 96% (146). Earlier work from the same group had already demonstrated that tumor-matched TP53 clonal variants could be identified in Pap test samples collected up to 68 months before HGSOC diagnosis (147). Although these findings are still preliminary and require prospective validation, they are particularly relevant to hereditary cancer settings, especially *BRCA1/2* carriers, in whom most ovarian cancers are high-grade serous and frequently arise from the distal fallopian tube. This approach therefore represents a promising, minimally invasive strategy for molecular interception of ovarian carcinogenesis before overt disease becomes clinically apparent.

To conclude, the convergence of liquid biopsy, immunopreventive vaccination, epigenetic biomarkers, early carcinogenesis markers, microbiota and AI-based analytics represents a paradigm shift in the management of HCS. These tools offer unprecedented opportunities to refine early detection strategies, tailor preventive interventions, and ultimately improve clinical outcomes in genetically predisposed individuals, provided that robust validation, ethical safeguards, and equitable implementation frameworks are established. Indeed, standardized assay platforms, rigorous cross-population validation, and extensive longitudinal clinical outcome data are required to ensure the clinical applicability of these tools in routine surveillance protocols for high-risk individuals. Addressing these challenges will be critical to translating technological innovations into meaningful improvements in clinical practice and health outcomes.

## 9 Conclusions

Preventing gynecologic cancers in women with HCS represents one of the most promising applications of precision medicine. Indeed, women who carry PVs in cancer predisposing genes, such as *BRCA1, BRCA2* and mismatch repair genes, face substantially elevated lifetime risks of ovarian and endometrial cancer compared with the general population, and these risks can be mitigated through a combination of genetic testing, individualized surveillance, prophylactic interventions, and evidence-based lifestyle guidance.

Prophylactic surgeries such as bilateral salpingo-oophorectomy and total hysterectomy with bilateral salpingo-oophorectomy remain the most effective strategies to reduce gynecologic cancer incidence in high-risk women, with substantial reductions in ovarian and breast cancer risk for *BRCA* carriers and endometrial and ovarian cancer for Lynch syndrome carriers, respectively. However, the timing and extent of prophylactic interventions must be carefully individualized, balancing oncologic benefit with psychosocial impact, reproductive goals, and quality of life, and supported by thorough genetic counseling (1, 55, 148). Post-surgical management, particularly following RRSO performed before natural menopause, demands attention to the sequelae of premature surgical menopause. Early loss of ovarian function is associated with increased risks to bone, cardiovascular, cognitive, and sexual health, and HRT, when appropriately selected, can mitigate many of these consequences without substantially increasing cancer risk in selected populations (100).

Fertility preservation and reproductive planning are key for carriers of high-penetrance variants. Preimplantation genetic testing enables informed reproductive choices and can prevent transmission of PVs, but psychosocial and ethical considerations remain central to counseling and decision-making (45, 46).

Despite these advances, significant disparities persist in access to genetic testing and preventive care. Socioeconomic status, geographic location, and racial and ethnic background continue to influence the likelihood that women at risk will receive timely and appropriate interventions. Addressing these inequities requires systemic changes in healthcare delivery, expanded access to genetic counseling, and culturally competent outreach within at-risk communities (149).

Finally, the lack of robust screening modalities for many hereditary cancers, particularly OC, underscores the urgent need for innovation. Conventional imaging and biomarker approaches have limited sensitivity in early disease, and emerging technologies such as liquid biopsy, methylation-based assays, and AI-driven predictive models hold promise for earlier detection and improved risk stratification. Investment in translational research and longitudinal cohort studies will be essential to validate these tools and confirm their impact on clinical outcomes (150, 151). Moreover, additional studies are warranted to define how modulation of the gut microbiota and the development of targeted vaccines may contribute to cancer prevention in HCS carriers.

In summary, the future of hereditary cancer management lies in a personalized, equitable, and evidence-based approach that integrates genetic risk with clinical context, patient values, and technological innovation. Only through such a comprehensive framework will be possible to improve outcomes and empower individuals at inherited risk of cancer, fulfilling the promise of precision prevention in clinical care.
